# Changes in the prevalence of measures associated with hypertension among Iranian adults according to classification by ACC/AHA guideline 2017

**DOI:** 10.1186/s12872-020-01657-0

**Published:** 2020-08-14

**Authors:** Mohsen Mirzaei, Masoud Mirzaei, Mojtaba Mirzaei, Behnam Bagheri

**Affiliations:** 1 Yazd Cardiovascular Research Center, Shahid Sadoughi University of Medical Sciences, Yazd, Iran; 2 Yale New Haven Medical Center, Waterbury Hospital, Waterbury, USA; 3 Shahediah Cohort Study, Shahid Sadoughi University of Medical Sciences, Yazd, Iran

**Keywords:** Hypertension, Prevalence, 2017 ACC/AHA hypertension guideline

## Abstract

**Background:**

Different definitions have been proposed to categorize hypertension. We aimed to investigate the difference in prevalence of measures associated with hypertension according to the American College of Cardiology/American Heart Association (ACC/AHA) criteria versus Joint National Committee 7 (JNC7) criteria.

**Methods:**

We analyzed the data of 10,000 participants of Yazd Health Study (YaHS) aged 20–69 years. Blood pressure was measured three times with standard protocol defined by ACC/AHA. Prevalence of high blood pressure measure was compared in both definitions and absolute differences reported.

**Results:**

The prevalence of high blood pressure in our measurement was 61.0% according to ACC/AHA, and 28.9% according to JNC 7. The prevalence of self-reported hypertension was 18.6%. Age and sex standardized prevalence rates of high blood pressure measure indicates a 2.4-fold increase in the prevalence rate (30.1% absolute difference) by the ACC/AHA guideline. While the prevalence increased in all age groups, the age group of 20–29 showed the highest relative increase by 3.6 times (10.6% vs. 38.1%). High blood pressure measure among people with diabetes increased from 45.8 to 75.3% with the ACC/AHA guideline. Of the people who had no past history of diagnosed hypertension (*n* = 7887), 55.1 and 22.7% had high blood pressure measure by ACC/AHA and JNC-7 guidelines, respectively. From JNC7 to ACC/AHA, the overall difference in unawareness about HTN increased by 32.4%.

**Conclusion:**

Prevalence of hypertension associated measures increased over two folds by using the ACC/AHA criteria compared to JNC 7. Also, change in the criteria, reduces awareness of the disease and increases uncontrolled hypertension respectively. More research is needed to determine if the new definitions can affect management of hypertension in societies. Considering local priorities and implication of cost effective may improve implementation of new definitions for hypertension in different countries.

## Background

Hypertension (HTN) is a global public health crisis; it is the most important risk factor for people with cardiovascular disease, which is one of the main causes of death and life lost in the world [1]. The prevalence of HTN was reported 20–50% across different world regions due to its differences in environmental and genetic factors and also changes in the study protocol [2]. In Asia, the prevalence was estimated 15–35%, in the Eastern Mediterranean Region (EMR) 29% and in Iran 18–23% [3–5]. In 2017, High blood pressure was the second most common risk factor for men and the first common risk factor for women [6]. In the past decades, due to the rapid socio-economic and lifestyle changes, the prevalence of non-communicable diseases such as hypertension has been increased in developing countries more than developed countries (7.7% compared to 2.6%) [7]. Blood pressure over 120/80 mmHg is linearly associated with the risk of cardiovascular disease, with an increase of 20 mmHg in systolic blood pressure and 10 mmHg in diastolic blood pressure, increase doubled the risk [8].

In 2017, the American College of Cardiology/American Heart Association (2017 ACC/AHA) guideline introduced a new classification for high blood pressure in adults which was a change in the clinical hypertension guide since 2003. In the ACC/AHA definition, with a decrease of 10 mmHg, the systolic blood pressure ≥ 130 and diastolic ≥80 mmHg are considered hypertension. In this guide, instead of the “pre-hypertension” category, a new group named “elevated hypertension” has been introduced [9]. According to this ACC/AHA guideline, people who have previously been classified as pre-hypertensive will be considered hypertensive. This new definition aimed at persuading patients to change lifestyles and use medications to reduce blood pressure. Early intervention for the care and control of hypertension reduces mortality and morbidity of cardiovascular disease [9].

Due to the difference in the prevalence of the disease in different regions, this new approach has prompted researchers to update their findings from surveillances of hypertension. Muntner and colleagues show that the prevalence of hypertension among Americans over 20 years of age increases by 14.7%, according to the ACC/AHA definition [10]. Another study shows a relative increase in the prevalence of 45.1 and 26.8% of the disease among 45–75 years-old in the United States and China [11]. Therefore, a significant number of adults over the age of 20 in the United States are unaware of their hypertension, are not being treated, or are not controlled their blood pressure adequately. The number of these people are more than 50 million people or 1 in 4 people of all men and 1 in 5 of all women [12]. The study of Khera in Nepal shows a 23% increase in the prevalence of high blood pressure with the ACC/AHA definition [13]. In Bangladesh, the difference was reported as 22.3% [14].

The purpose of this study was to estimate the prevalence of hypertension associated measures in a large number of adults in an Iranian population according to the new guidelines (ACC/AHA 2017) and compare it with the old definition (JNC 7). The difference between the two guidelines in the prevalence of hypertension associated measures is a guide for health managers to revise intervention programs for disease control accordingly.

## Methods

Yazd Health Study (YaHS) is a prospective cohort study conducted since 2014 to determine determinants of non-communicable disease and related risk factors in Yazd Greater Area which is located in the center of Iran. Details of YaHS have been published elsewhere [15]. Briefly, 10,000 residents of Yazd Greater Area at the age of 20 to 69 years were selected using cluster random sampling method. Fifty people interviewed in each cluster with 50 people (25 males and 25 females), in which five individuals were selected from each age group.

A valid questionnaire was completed at home visits. In follow-up, the final overall response rate was 98%. The previous history of HTN diagnosis by a physician was documented according to self-report. Self-reported hypertension has been recorded with a positive answer to the question, “have you ever been told by your physician that you have high blood pressure?” An English version of the YaHS questionnaire has been published elsewhere [16]. Also, it can be accessed at the study website from the address: http://www.yahs-ziba.com. At the physical examination, three blood pressures (BP) were measured by trained and certified person at home visit (out-of-office method) using standard method with appropriate cuff sizes for participants’ arm [17]. Blood pressure was measured in a sitting position after two-thirds of the interview questions were completed, so the interviewees had been in rest for at least 40 min at the time of measurement. It was ensured that sphygmomanometer cuff, used to measure BP, was at the level of the heart and the cuff was deflated slowly. Blood pressure measurements were repeated three times with 5 min interval using electronic sphygmomanometers (Model N-Champion, Reichter GMBH, Germany) which were calibrated regularly. The mean of the second and third measurements was recorded as blood pressure, which was used for analysis [14, 15, 18, 19].

Measured BP was classified into normal, pre-hypertension, hypertension stage-1, and hypertension stage-2 by Joint National Committee (JNC 7) classification for adults [18]. The 2017 ACC/AHA guideline has changed the definition of high blood pressure and its classification; the definition of hypertension was systolic BP ≥130 mmHg and/or a diastolic BP of ≥80 mmHg. In other words, the cut-off point reduced by 10 mmHg for systolic BP and 5 mmHg for diastolic BP. Additionally, the definition of pre-hypertension has been changed and a new class called elevated blood pressure has been introduced. Therefore, the definition of stage-1 and stage-2 hypertension has undergone a change. The Table 1 shows the comparison of hypertension classification by JNC 7 and ACC/AHA [19].

**Table 1:** Blood Pressure classifications by JNC 7 and 2017 ACC/AHA guideline

Systolic BP (mm Hg)		Diastolic BP (mm Hg)	JNC 7^a^	2017 ACC/AHA^b^
< 120	And	< 80	Normal	Normal
120–129	And	< 80	Pre-hypertension	Elevated BP
130–139	Or	80–89	Pre-hypertension	hypertension-Stage 1
140–159	Or	90–99	hypertension-Stage 1	hypertension-Stage 2
≥ 160	or	≥ 100	hypertension-Stage 2	hypertension-Stage 2

^a^Seventh Report of the Joint National Committee

^b^ 2017 American College of Cardiology (ACC)/ American Heart Association (AHA) guideline

Body Mass Index (BMI) calculated as weight/height^2^ in kg/m^2^ and was categorized to; underweight, normal, overweight, and obese according to the World Health Organization (WHO) cut-off point’s recommendation [20].

Mean BP level values ±standard deviations (SD) were calculated by demographic data and past medical history. The prevalence of hypertension associated measures was described as proportions with 95% confidence intervals (CI). Age-standardized prevalence rates were calculated using the direct method according to 2016 Yazd population census. The absolute differences in prevalence of high blood pressure measures according to guidelines were calculated separately. A chi-square test was used to detect the significance of change. All statistical analyses were performed using SPSS version 16 software. A *p*-value less than 0.05 were considered statistically significant.

The research proposal was approved by the ethics committee of Shahid Sadoughi University of Medical Science, Yazd, Iran No: IR.SSU.MEDICINE.REC.1396.311. The study was explained to all respondents willing to participate. All participants had the right to withdraw from the study at any time. Informed consent was obtained from each participant before data collection. Participants with new diagnostics of hypertension were advised to refer their health center or physician for the follow-up.

## Results

In this study, the blood pressure of 9861 adults was measured three times. Of total, 18.6% had self-reported hypertension. The crude prevalence of high blood pressure associated with HTN according to JNC 7 guideline was 28.9% (CI 95%: 27.9–29.7), and according to the ACC/AHA 2017 was 61.0% (95% CI: 60.1–62.0%); an absolute increase of 32.1%. Age and sex standardized prevalence rates of high blood pressure was 21.6 and 51.7% according to JNC7 guideline and ACC/AHA 2017, respectively. This indicated a 2.4-fold increase in the prevalence rate accordingly (30.1% absolute difference). Of the people who had no past history of hypertension (*n* = 7887), 22.7 and 55.1% had high blood pressure by JNC-7 and ACC/AHA 2017 guidelines, respectively who were not aware of their illness and were not being treated.

Two-thirds of men have blood pressure measure in the range of HTN according to ACC/AHA guideline (compared to 32.5% according to the previous classification). This increased absolute prevalence is higher in men compared with women (35.0% vs. 29.5%). With aging, the prevalence of hypertension associated measures increased, but the highest absolute increase was seen in the group 40–49 years old with the ACC/AHA definitions (35%). In the age of 20–29 years, the prevalence of high blood pressure measurement increased approximately 4 times (10.6% vs. 38. 1%).With the ACC/AHA definition, about 69% of obese people have high blood pressure by ACC/AHA 2017 guideline, compared with 38.6% by JNC 7. With the new guideline, three out of four diabetic patients were in the hypertensive group, which shows an absolute increase of 30% (Table 2).

**Table 2:** Characteristics of the Yazd Health Study participants (20–69 years) and the prevalence of hypertension associated measures by both guidelines in 2014–15

Variables	Total Num. (%)	Prevalence of hypertension associated measures		Difference %
		JNC-7 Num. (%, CI 95%)	ACC/AHA2017 Num. (%, CI 95%)	
Sex				
Male	4883 (49.5)	1585 (32.5,31.2–32.5)	3152 (66.5,63.2–65.9)	+ 34.0
Female	4978 (50.5)	1275 (25.6,24.4–26.8)	2617 (53.8,52.4–55.2)	+ 28.2
Age groups				
20–29	1952 (19.8)	206 (10.6,9.3–12.0)	737 (38.1,36.0–40.3)	+ 27.5
30–39	2015 (20.5)	355 (17.6,16.0–19.3)	1026 (51.4,49.2–53.6)	+ 33.8
40–49	2042 (20.7)	592 (28.9,27.1–31.0)	1273 (64.0,61.9–66.1)	+ 35.1
50–59	1957 (19.8)	724 (37.0,34.9–39.2)	1338 (70.3,68.2–72.3)	+ 33.3
60–69	1893 (19.2)	965 (50.9,48.7–53.2)	1394 (78.0,76.0–79.9)	+ 27.1
Education				
Primary school and less	2550 (26.1)	1089 (42.7,40.8–44.6)	1784 (73.1,71.3–74.7)	+ 29.4
High school	2783 (28.5)	808 (29.0,27.4–30.7)	1688 (62.1,60.2–63.9)	+ 33.1
Diploma and Graduate Diploma	2899 (29.7)	624 (21.5,20.1–23.0)	1521 (53.4,51.5–55.2)	+ 31.9
BSc,MSc. and Doctorate	1535 (15.7)	296 (19.3,17.4–21.3)	725 (48.0,45.5–50.5)	+ 28.7
BMI (kg/m2)				
Underweight (< 18.5)	299 (3.0)	27 (9.0,6.3–12.8)	80 (26.9,22.2–32.3)	+ 17.9
Normal (18.5–24.5)	3149 (32.0)	614 (19.5,18.1–20.9)	1520 (49.2,47.5–51.0)	+ 29.7
Overweight (25.0–29.9)	3796 (38.6)	1193 (31.4,29.9–32.9)	2368 (64.1,62.5–65.6)	+ 32.7
Obesity (≥30.00)	2601 (26.4)	1003 (38.6,36.7–40.4)	1795 (71.4,69.6–73.1)	+ 32.8
History of hypertension				
Yes	1800 (18.6)	1069 (59.4,57.1–61.4)	1402 (83.6,81.7–85.3)	+ 24.2
No	7887 (81.4)	1743 (22.1,21.2–23.0)	4275 (55.1,54.0–56.2)	+ 33.0
History of diabetes mellitus? mellitus				
Yes	1386 (14.1)	635 (45.8,43.2–48.4)	999 (75.3,72.9–77.6)	+ 29.5
No	8420 (85.9)	2194 (26.1,25.1–27.0)	4732 (57.5,56.4–58.6)	+ 31.4

Table 2 shows the characteristics of the participants and comparison of hypertension associated measures according to both guidelines (JNC-7 & ACC/AHA 2017) in different groups. With the ACC/AHA 2017 was observed a crude increase of 24% or more in the prevalence of hypertension associated measures in all age-sex groups, education and people with a history of diabetes. This change is also seen in people who are overweight or obese; even with a normal body mass index. The highest increased proportion is seen in men 40–49 year’s age groups.

Mean systolic and diastolic BP in the population were 126.5 ± 18.4 mmHg and 80.2 ± 12.5 mmHg, respectively. Men had higher mean systolic and diastolic BP than women in the study participants**.** The mean systolic and diastolic BP increases with age, less education and higher body mass index. This trend is similar in both sexes. According to the ACC/AHA classification, 19.1% of the individuals with systolic HTN (130–139 mmHg) which corresponds to pre-hypertension in the previous definition were associated with hypertension-stage 1. For diastolic blood pressure, this difference was 33.7%.

Table 3 shows that the new classification of 61% of the adults according to systolic blood pressure and 48% according to diastolic blood pressure changed with the new definitions. This change can potentially initiate preventive and therapeutic interventions for these individuals as compared to the previous definition.

**Table 3:** Frequency distribution of blood pressure levels in adult residents of Yazd Greater Area aged 20–69 years in 2014–2015

Blood Pressure level (mm Hg)	Male Num. (%, CI 95%)	Female Num. (%, CI 95%)	Total Num. (%, CI 95%)
Systolic BP			
< 120	1278 (26.2,25.0–27.4)	1987 (39.9,38.6–41.3)	3265 (33.1,32.2–34.0)
120–129	1412 (28.9,27.7–30.2)	1227 (24.6,23.5–25.9)	2639 (26.8,25.9–27.6)
130–139	1071 (21.9,20.8–23.1)	816 (16.4,15.4–17.4)	1887 (19.1,18.4–19.9)
140–149	600 (12.3,11.4–13.2)	448 (9.0,8.2–9.8)	1048 (10.6,10.0–11.2)
150–159	215 (4.4,3.9–5.0)	217 (4.4,3.8–5.0)	432 (4.4,4.0–4.8)
≥ 160	307 (6.3,5.6–7.0)	283 (5.7,5.1–6.4)	590 (6.0,5.5–6.5)
	*P* value: < 0.0001		
Diastolic BP			
≤ 79	1954 (40.0,38.6–41.4)	2638 (53.0,51.6–54.4)	4592 (46.6,45.6–47.5)
80–89	1797 (36.8,35.5–38.2)	1523 (30.6,29.3–31.2)	3320 (33.7,32.7–34.6)
90–99	798 (16.3,15.3–17.4)	597 (12.0,11.1–12.9)	1395 (14.1,13.5–14.8)
≥ 100	334 (6.8,6.2–7.6)	221 (4.4,3.9–5.1)	555 (5.6,5.2–6.1)
	P value: < 0.0001		

**Fig. 1:**
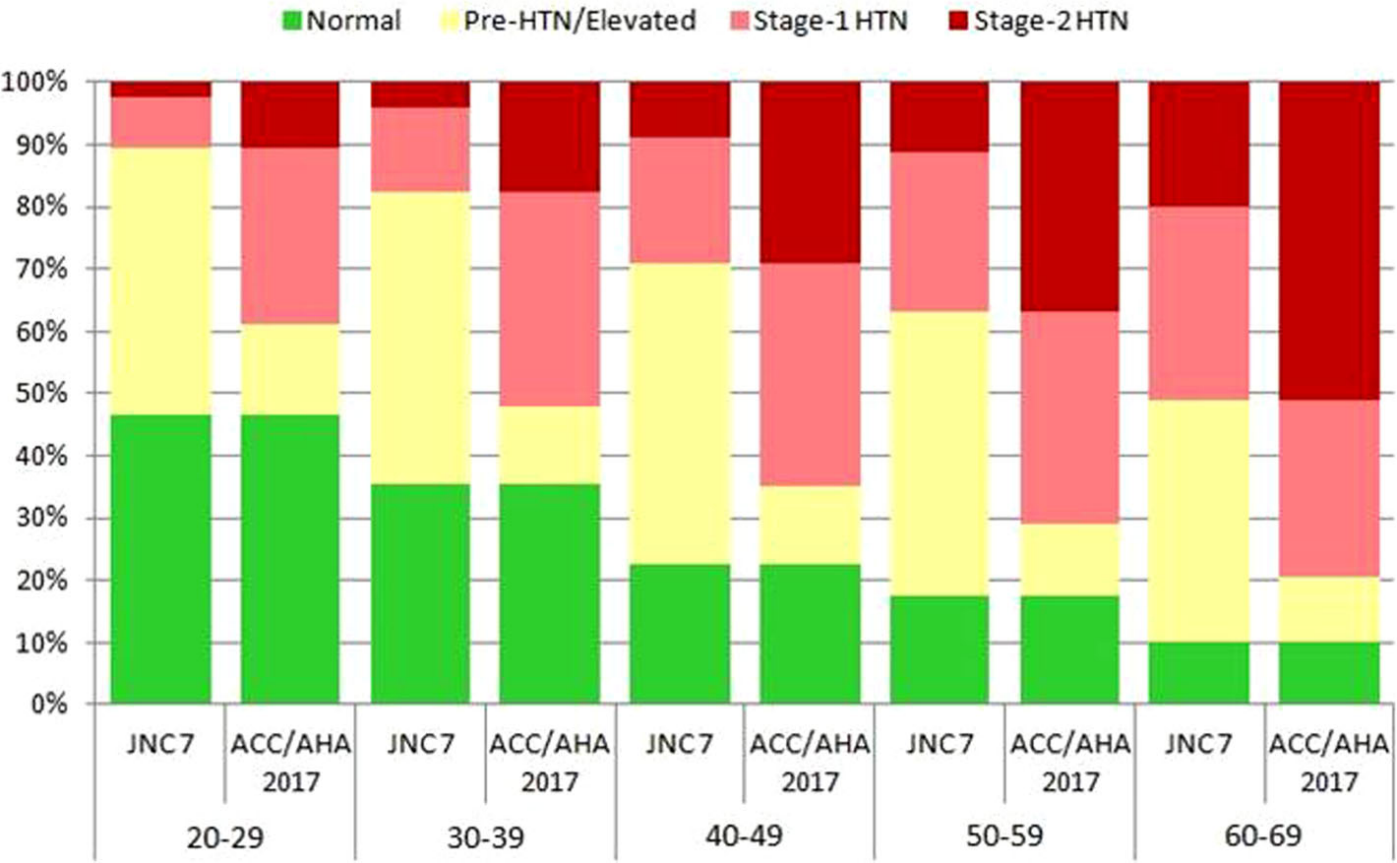
Prevalence of hypertension associated measures in various age groups by JNC-7 and ACC/AHA 2017 guidelines

The 2017 ACC/AHA guideline was associated with an increase in most of the HTN categories in our study. Men had a higher absolute increase in prevalence than women. A similar increase was observed for hypertension stage 1 and 2 for both sexes. But the increase in hypertension-stage 2 was higher in men than in women (22.1% vs. 17.1%). Table 4 shows the difference in the stages of the two definitions of hypertension by sex. This increase in the percentage of hypertension-stage 2 was also seen in different age groups. As shown in Fig. 1, this difference is more pronounced in younger adults and may cause interventions to begin at a lower age.

**Table 4:** Comparison of hypertension associated measures in males and females residents of Yazd Greater area according to JNC7 and ACC/AHA 2017 guidelines 2014–2015

Hypertension Prevalence by BP measurement	JNC 7 Num. (%, CI 95%)	ACC/AHA2017 Num. (%, CI 95%)	Difference %
**Male (N:4883)**			
Normal	978 (20.0,18.9–21.2)	978 (20.0,18.9–21.2)	**0**
Pre-hypertension/elevated blood	2320 (47.5,46.1–48.9)	613 (12.6,11.6–13.5)	**- 34.9**
Stage-1 hypertension	1075 (22.0,20.9–23.2)	1707 (35.0,33.6–36.3)	**+ 13**
Stage-2 hypertension	510 (10.4,9.6–11.3)	1585 (32.5,31.2–33.8)	**+ 22.1**
Hypertension (stage-1 plus stage-2)	1585 (32.4,31.2–33.8)	3292 (67.4,66.1–68.7)	**+ 35.0**
**Female (N:4978)**			
Normal	1642 (33.0,31.7–34.3)	1642 (33.0,31.7–34.3)	**0**
Pre-hypertension/elevated blood	2079 (41.8,40.4–43.1)	608 (12.2,11.3–13.1)	**−29.6**
Stage-1 hypertension	854 (17.2,16.1–18.2)	1471 (29.6,28.3–30.8)	**+ 12.4**
Stage-2 hypertension	403 (8.1,7.4–8.9)	1257 (25.2,24.1–26.5)	**+ 17.1**
Hypertension (stage-1 plus stage-2)	1257 (25.3,24.1–26.5)	2728 (54.8,53.4–56.2)	**+ 29.5**
**Total**			
Normal	2620 (26.6,25.7–27.4)	2620 (26.6,25.7–27.4)	**0**
Pre-hypertension/elevated blood	4399 (44.6,43.6–45.6)	1221 (12.4,11.7–13.0)	**−32.2**
Stage-1 hypertension	1929 (19.6,18.8–20.3)	3178 (32.2,31.3–33.2)	**+ 12.6**
Stage-2 hypertension	913 (9.3,8.7–9.8)	2842 (28.8,27.9–29.7)	**+ 19.5**
Hypertension (stage-1 plus stage-2)	2842 (28.9,27.9–29.7)	6020 (61.0,60.1–62.0)	**+ 32.1**

## Discussion

About 60% of the study populations had blood pressure associated with HTN according to AHA/ACC guideline, about twice the prevalence by JNC-7 threshold, however the ratio of men to women with hypertension remains almost the same according to either guideline (1.3 vs. 1.2). More than half of the participants with hypertension who did not have any history of diagnosed hypertension will have high blood pressure according to the ACC/AHA guideline. High blood pressure was more prevalent among males and those with lower education and higher BMI.

In a study by Esteghamati et al. a similar hypertension prevalence of 25.6% among Iranian adults according to JNC-7 criteria was reported [21]. However, another study of the rural population in Iran showed a prevalence of 41.8% [22]. as expected, the prevalence of hypertension will increase according to AHA/ACC guideline with low thresholds. The amount of this increase was not the same among different studies in Iran and other countries. While we reported an increase of over two folds, another study in Iran found an increase of about three folds from 12.6 to 42.7% from the fifth phase (2012–2015) of the Tehran Lipid and Glucose Study [23]. An increase of 1.4 times from 31.9 to 45.6% in United States, [10] 1.6 times from 30.4 to 49.2% in Korea [24] and 1.9 times in Southwest China [25] was seen in different populations across the world.

According to the AHA/ACC guideline, the prevalence of high blood pressure increased in all age groups but the difference was more prominent in younger age groups in our study. This should not be mixed-up with indication to treatment, as the pharmacologic management of hypertension in the elderly population is the main expected change according to the ACC/AHA guideline. Among those 60–69 years old, 78% had high blood pressure according to ACC/AHA guidelines in our study; this was 51% according to JNC-7. The difference between these two figures may indicate the need for mass medications in the population. While JNC-8 guidelines were considered an easier standard for treatment in those older than 60 years compared to JNC-7, [26] the ACC/AHA guideline introduced a stricter target.

The prevalence of high blood pressure among patients with diabetes in our study increased with the ACC/AHA guidelines from 45.8 to 75.3%. However, any benefit from the intervention in a larger population from this new classification remains questionable. The American diabetes association still recommends blood pressure threshold of 140/90 mmHg for initiation of pharmacologic therapy which does not agree with the general target of 130/80 for diabetic patients [27]. While one of the main studies behind the AHA/ACC recommendation is the SPRINT trial, those with diabetes were excluded from that study [28]. ACCORD trial in patients with hypertension and diabetes showed no additional benefit of treating blood pressure to 120 mmHg compared to 140 mmHg in those with diabetes [29].

High blood pressure according to the ACC/AHA guideline does not mean an indication for pharmacotherapy. Three months’ trial of lifestyle modification is needed for most of those labeled ‘hypertensive’ according to the new guidelines [9]. Although this difference can change the prevalence of patients labeled with HTN considerably, a study by Muntner et al. [10] showed that the overall impact on indications for treatment will be much less. We were not being able to calculate all the people who need to start pharmacologic therapy according to the ACC/AHA guidelines.

Although the AHA/ACC updated its threshold for HTN, many other American and European associations have not updated their guidelines accordingly. European Society of Cardiology and European Society of Hypertension in their updated guidelines defined hypertension the same as JNC-7 [30]. ‘American Academy of Family Physicians’ and ‘American College of Physicians’ still recommend the JNC 7 thresholds and published their guidelines for treatment of hypertension in elderly, accordingly [31]. Multiple thresholds for hypertension and its management have the potential to confuse clinicians and public health policymakers when comparing data from different countries or different time periods.

There is controversy about the negative effects of labeling patients with hypertension. Some studies have reported negative outcomes including distress and higher levels of physical symptoms, increased work absenteeism and lower health-related quality of life, [32–34] while others have shown no negative effect [35]. In addition to this controversy, there is no clear evidence that changing HTN threshold will change outcomes. Similar worries arose when introducing the term of “pre-hypertension” in previous guidelines. A study reported no such changes in eating habits, salt intake, alcohol drinking and exercise [36].

In addition to changing hypertension definition, ACC/AHA guidelines lowered targets for blood pressure management, especially in older population. Pharmacotherapy for lowering blood pressure below 130/80 mmHg is now recommended in general population. This may have significant impact, especially in older population. It should be noted that JNC 8 panel members report increased target of blood pressure therapy in those older than 60 years to 150 mmHg which is 20 mmHg higher than current recommendation.

In the past decades, despite an increase in the prevalence of hypertension, control of this condition was improved in the United States [37]. Using the AHA/ACC criteria, temporal comparison of hypertension awareness and management will be more difficult especially in developing countries with limited availability data. In addition to difficulty in temporal comparison, regional comparison will be difficult considering the responsible authority. Even according to the JNC guideline, a considerable proportion of population was unaware of their hypertension status. The proportion will be increased more than twice by application of the new AHA/ACC guideline.

Although there is controversy about the new definition of hypertension among medical authorities around the world, the threshold of normal blood pressure and recommendations for medical treatment is much more similar. Different terms for describing hypertension levels such as “elevated”, “pre-hypertension” and “stage 1 hypertension” may cause confusion for providers and patients. The use of similar and equivalent terms can simplify the doctor-patient communication and facilitate the analysis of public health trends over time. Non-medical management of high blood pressure should be tailored to the culture of local communities and not necessarily limited to the specific terms for describing high blood pressure.

### Limitation

This is a cross-sectional study with three consequent BP measurements with 5 min’ interval. Although measuring blood pressure in two occasions was recommended to label patients with hypertension, we only measured blood pressure in 1 day for this study due to the large sample size and logistic issues. However, we tried to increase accuracy by repeating the measurement and report the average of the last two times similar to other large epidemiologic studies. Inter-observer differences in measuring blood pressure can lead to misclassification. However, our interviewers were trained in practical courses and digital calibrated sphygmomanometer was used with minimum interpretation by the interviewers.

## Conclusion

This study shows that the ACC/AHA guideline puts the blood pressure of a significant proportion of adults in the range of hypertension. This public health problem calls for more comprehensive interventions to prevent and control hypertension. Changes in HTN definition may prompt people to change their lifestyles sooner and further investigation into the initiation of medical treatment is warranted. In addition, differences in the prevalence of cardiovascular risk factors, unhealthy lifestyle, and health care systems in countries should be considered in determining cut-off points for initiating treatment. Prospective research is useful to evaluate the effect of this change in the guideline on the rate of uncontrolled high blood pressure complications.

## Data Availability

The data collected by Yazd Health Study are not open access but can be shared under conditions of collaboration and endowment. Data are available from the authors upon reasonable request and with permission of principal investigator. For further information, please visit YaHS website at www.yahs-ziba.com / www.yahs.ir.

## Abbreviations

ACC/AHA: American College of Cardiology/American Heart Association
JNC7: Joint National Committee 7
YaHS: Yazd health study
HTN: Hypertension
EMR: Eastern Mediterranean region
WHO: World Health Organization
BMI: Body mass index
